# The relationship of lung function with ambient temperature

**DOI:** 10.1371/journal.pone.0191409

**Published:** 2018-01-18

**Authors:** Joseph M. Collaco, Lawrence J. Appel, John McGready, Garry R. Cutting

**Affiliations:** 1 Johns Hopkins University School of Medicine, Baltimore, Maryland, United States of America; 2 Johns Hopkins Bloomberg School of Public Health, Baltimore, Maryland, United States of America; Central Michigan University College of Medicine, UNITED STATES

## Abstract

**Background:**

Lung function is complex trait with both genetic and environmental factors contributing to variation. It is unknown how geographic factors such as climate affect population respiratory health.

**Objective:**

To determine whether ambient air temperature is associated with lung function (FEV_1_) in the general population.

**Design/Setting:**

Associations between spirometry data from two National Health and Nutrition Examination Survey (NHANES) periods representative of the U.S. non-institutionalized population and mean annual ambient temperature were assessed using survey-weighted multivariate regression.

**Participants/Measurements:**

The NHANES III (1988–94) cohort included 14,088 individuals (55.6% female) and the NHANES 2007–12 cohort included 14,036 individuals (52.3% female), with mean ages of 37.4±23.4 and 34.4±21.8 years old and FEV_1_ percent predicted values of 99.8±15.8% and 99.2±14.5%, respectively.

**Results:**

After adjustment for confounders, warmer ambient temperatures were associated with lower lung function in both cohorts (NHANES III *p* = 0.020; NHANES 2007–2012 *p* = 0.014). The effect was similar in both cohorts with a 0.71% and 0.59% predicted FEV_1_ decrease for every 10°F increase in mean temperature in the NHANES III and NHANES 2007–2012 cohorts, respectively. This corresponds to ~2 percent predicted difference in FEV_1_ between the warmest and coldest regions in the continental United States.

**Conclusions:**

In the general U.S. population, residing in regions with warmer ambient air temperatures was associated with lower lung function with an effect size similar to that of traffic pollution. Rising temperatures associated with climate change could have effects on pulmonary function in the general population.

## Introduction

Lung function is complex trait with both genetic and environmental factors contributing to its variation.[1–4] A number of environmental factors that impact lung function on an individual level have been described, such as indoor combustion,[5] environmental tobacco smoke,[6] and proximity to traffic.[7–9] Air pollution is one of the few population-wide environmental factors that have been shown to affect lung function in large numbers of individuals simultaneously.[8,10] Ambient air temperature is another large-scale geographic factor which may affect lung function, and respiratory health overall, that may have added relevance given rising temperatures associated with climate change.

Temperature has been recognized as a risk factor for worse outcomes in respiratory diseases.[11] Notably, acute respiratory events and exacerbations of existing respiratory disease are more likely to occur during times of temperature extremes. With heat waves and cold snaps, an increased frequency of exacerbations and/or deaths has been observed in asthma and COPD.[12–14] Potential long-term effects of ambient temperature on lung function are less well studied. We have previously demonstrated an association between lower lung function (FEV_1_) in individuals with cystic fibrosis and warmer annual ambient temperatures with replication in two national cystic fibrosis registries in the United States and Australia.[15] The observed association in cystic fibrosis was only partially explained by the geographic distribution of common respiratory pathogens in cystic fibrosis,[15–17] suggesting that other factors play a role.

In this context, we speculated that mean annual ambient temperatures may be associated with lung function in individuals in the general population. To test this hypothesis, we obtained lung function (FEV_1_) data in healthy non-smokers in the United States from two separate cohorts from the National Health and Nutrition Examination Survey (NHANES) and tested for association with mean annual ambient temperatures.

## Methods

### Study population

De-identified data were utilized from the National Health and Nutrition Examination Survey (NHANES) conducted by the National Center for Health Statistics (NCHS). NHANES is a series of surveys that collect questionnaire and medical testing data on a sample of the non-institutionalized U.S. population. All surveys where spirometry was routinely performed were used, including the periods 1988–1994 (NHANES III), 2007–08, 2009–10, and 2011–12. The NHANES 2007–08, 2009–10, and 2011–12 data were combined for analysis as they utilize similar survey weights. To obtain a sample of participants in good respiratory health, participants were excluded if they had any mention of asthma or any reported active tobacco product use. Of note, specific questions for COPD were not asked of the study populations. This study was exempt from IRB review under the guidelines of the Johns Hopkins University IRB as all data were de-identified and the investigators had no access to identifiable information, including location or date of birth.

### Demographic data

Raw data for sex, age, race/ethnicity, annual household income, and insurance status were all obtained from NHANES publically available datafiles. Any participant reporting any non-white race or Hispanic ethnicity was coded as non-white for the purposes of regression modeling. Participants who had insurance were coded as either having no insurance, public insurance, or private insurance with any mention of private insurance being coded as private for the purposes of regression modeling.

### Lung function data

Lung function was collected by trained personnel in a standardized approach in accordance with American Thoracic Society guidelines.[18,19] Raw forced expiratory volume in 1 second (FEV_1_) in milliliters was converted to percent predicted values using NHANES reference equations.[20,21] Participants without valid FEV_1_ data were excluded. Ambient temperature measurements in the testing facility at the time of testing were available only for the NHANES III cohort.

### Temperature data

Temperature source data consisted of a 30 year average of ambient temperature (1980–2010; The Climate Source, Inc.; Corvallis, OR) to minimize year-to-year variation in annual temperatures. Participants’ reported residential 5-digit postal zip code were merged with a file containing the mean annual ambient temperature by population centroid of all U.S. postal zip codes mapped using ESRI ArcGIS (ESRI; Redlands, CA).

### Statistical analyses

Student’s *t* tests and chi-square tests were used to compare demographic factors between included and excluded participants. Linear regression was performed to determine if temperature was associated with lung function using separate models for the NHANES III data and the NHANES 2007–2012 data. The final models were adjusted for demographic factors that were associated with lung function in univariate regression. Although participants are selected to represent the U.S. population at all ages, NHANES routinely oversamples persons aged 60 years and older, African-Americans, and Hispanics; thus all regressions were survey weighted per recommended NHANES analytic practices.[22] A pooled analysis weighted by study n was used to provide a combined estimate of effect size from both NHANES datasets.[23] All statistical analyses were conducted using STATA IC 11 (College Station, TX) at the NCHS RDC facility (Hyattsville, MD) owing to the use of restricted data (zip code). A *p* value of <0.05 was considered statistically significant.

## Results

### Demographics

The NHANES III and NHANES 2007–12 survey periods consisted of 25,733 and 25,405 participants over 6 years of age who could have had spirometry measurements. After applying exclusion criteria, our study cohorts included 14,088 and 14,036 participants for the NHANES III and NHANES 2007–12 periods, respectively. Most were excluded because spirometry was not performed or the individual was an active tobacco user (S1 Fig).

Comparisons between our study cohorts and the excluded participants revealed that included participants were more likely to be female, younger, report higher household incomes, have private health insurance, reside in locations with warmer mean annual ambient temperatures, and have higher lung function (S1 Table). Of note, the percent predicted FEV_1_ was 6.3 (*p*<0.001) and 5.5 (p<0.001) percent predicted lower in those reporting tobacco use compared to those who did not in the NHANES III and NHANES 2007–12 populations, respectively. Other observed differences observed between our study cohort and excluded participants are likely related to the exclusion of participants with active tobacco use, which was more common among males, Blacks and Whites (less common in other racial/ethnic groups), older participants, and participants with lower reported incomes in both the NHANES III and NHANES 2007–2012 survey periods.

Survey weighted means for selected demographic variables, which account for NHANES oversampling of certain demographic groups, are reported in Table 1. These survey weighted means are more representative of the analytic cohorts than the raw data presented in eTable 1 as all further analyses are survey weighted in accordance with recommended practices for NHANES data.[22] Of note, our survey weighted cohorts are still more likely to be female and have higher household incomes than the reported U.S. means (~$30,000 in 1990 and ~$50,000 in 2010). Lung function (FEV_1_) approximated 100% of predicted in both study cohorts.

**Table 1:** Survey weighted means (± S.E.) for study cohorts.

Mean ± S.E.	NHANES III Cohort (n = 14,088)	NHANES 2007–2012 Cohort (n = 14,036)
**Sex** (% female)	54.5	53.2
**Race/Ethnicity** (%)		
Black	11.3	11.0
Hispanic	11.4	16.8
White	73.8	64.8
Mixed/Other	3.4	7.4
**Age** (years)	37.7 ± 0.4	37.6 ± 0.4
**Income** ($’000s)	31.7 ± 0.5 (n = 12,815)	59.9 ± 1.0 (n = 13,452)
**Insurance** (%)		
None	11.1	15.8
Private	66.8	66.2
Public	22.1 (n = 13,497)	18.1 (n = 14,008)
**Temperature** (°F)	57.1 ± 0.6	56.8 ± 0.5
**FEV**_**1**_ (% Predicted)	98.8 ± 0.3	98.7 ± 0.2

### Potential confounders

Survey-weighted univariate modeling of the association of lung function and selected demographic factors was performed to identify potential confounding factors (Table 2). In both study cohorts, most potential confounders, e.g. age, were similar; however, private insurance was associated with higher lung function only in the NHANES III cohort, while female sex was associated with higher lung function only in the NHANES 2007–2012 cohort. For the multivariate regression analyses that follow, all of these significant factors were included as covariates in the analysis of both study cohorts for consistency.

**Table 2:** Survey weighted univariate regressions.

Coefficient (change in FEV_1_% predicted) [95% C.I.]	NHANES III Cohort		NHANES 2007–2012 Cohort	
	n = 14,088	Coefficient *P* value	n = 14,036	Coefficient *P* value
**Sex** (male = 0; female = 1)	0.17 (-0.50, 0.84)	0.61	1.06 [0.38, 1.73]	0.003
**Race/Ethnicity** (white = 0; nonwhite = 1)	0.08 [-0.94, 1.11]	0.87	-0.29 [-0.98, 0.41]	0.41
**Age** (per year)	-0.12 [-0.14, -0.10]	<0.001	-0.14 [-0.16, -0.12]	<0.001
**Log Income** (per Log $)	0.74 [-0.08, 1.55] (n = 12,815)	0.08	0.48 [-0.68, 1.63] (n = 13,452)	0.41
**Insurance** (private = 0; public = 1)	-3.21 [-3.92, -2.50] (n = 11,088)	<0.001	0.76 [-0.18, 1.69] (n = 11,274)	0.11

### Relationship of lung function with temperature

In survey weighted, multivariate regression analyses incorporating the covariates identified in univariate analyses, higher mean annual ambient temperatures were associated with lower lung function in both the NHANES III study cohort *(p* = 0.020) and the NHANES 2007–2012 study cohort (*p* = 0.014)(Table 3). The magnitude of the effect was similar in both cohorts with a 0.71 and 0.59 percent predicted FEV_1_ decrease for every 10°F increase in mean annual ambient temperature in the NHANES III and NHANES 2007–2012 study cohorts, respectively. Pooled analysis incorporating the results from both study cohorts yielded an estimated effect size of 0.65 percent predicted decrease for every 10°F increase (95% C.I.: 0.3%, 1.0%; *p* = 0.001).

**Table 3:** Survey weighted multivariate regressions unadjusted and adjusted for potential confounders for study population.

Coefficient (change in FEV_1_% predicted) [95% C.I.]	NHANES III Cohort		NHANES 2007–2012 Cohort	
	n = 14,088	Coefficient *P* value	n = 14,036	Coefficient *P* value
**Temperature** (per 10°F) [UNADJUSTED]	-0.51 [-1.22, 0.19]	0.15	-0.46 [-0.91, -0.02]	0.042
**Temperature** (per 10°F) [ADJUSTED]*	-0.71 [-1.30, -0.11] (n = 11,088)	0.020	-0.59 [-1.06, -0.12] (n = 11,274)	0.014

*Adjusted for sex (male = 0; female = 1), age (years), and insurance status (private = 0; public = 1).

### Spirometry temperature

Given that changes in spirometry have been reported with the experimental exposure of exercising subjects to different temperatures,[24] we assessed whether ambient indoor temperature at the time of spirometry affected the association between mean annual ambient temperature and lung function. The NHANES III survey period but not the NHANES 2007–2012 period included the temperature at which spirometry was conducted (Mean ± S.D.: 74.2±2.7°F; n = 14,080). To assess whether spirometry temperature was a better predictor of lung function than mean annual ambient temperature, we performed adjusted regression analysis as above, including both ambient temperature and spirometry temperature as independent variables (Table 4). In this analysis, ambient temperature remained a significant predictor of lung function (*p* = 0.015), regardless of whether spirometry temperature was included in the regression model. In addition, the magnitude of the association remained constant as well (0.71 percent predicted FEV_1_ decrease for every 10°F increase in mean annual ambient temperature), also suggesting that this association is not affected by the temperature during testing.

**Table 4:** Adjusted survey weighted multivariate regressions to assess the effect of spirometry temperature in the NHANES III cohort*.

n = 11,087	Spirometry (Indoor) Temperature		Mean Annual Ambient Temperature	
	Coefficient (change in FEV_1_% predicted) [95% C.I.]	Coefficient *P* value	Coefficient (change in FEV_1_% predicted) [95% C.I.]	Coefficient *P* value
**Regression of Spirometry Temperature only**	-1.80 [-3.66, 0.06]	0.06	Not Applicable	
**Regression of Mean Annual Ambient Temperature only**	Not Applicable		-0.71 [-1.30, -0.11] (n = 11,088)	0.020
**Regression Including Both Temperatures**	-1.81 [-3.67, 0.05]	0.06	-0.71 [-1.28, -0.14]	0.015

*All regressions adjusted for sex (male = 0; female = 1), age (years), and insurance status (private = 0; public = 1).

### Additional sensitivity analyses

To assess the sensitivity of the results with regard to smoking (current and former), several additional analyses were undertaken. First, survey weighted regressions demonstrated a consistent association between higher temperature and lower FEV_1_ in both NHANES populations (NHANES III *p* = 0.005; NHANES 2007–2012 *p* = 0.010) when both current and former smokers were excluded. Second, in survey weighted regressions including never, former and current smokers, there were no interactions between temperature and any history of smoking (NHANES III *p* = 0.11; NHANES 2007–2012 *p* = 0.62). Third, analyses were conducted also using smoking pack years (1 pack year = smoking 20 cigarettes daily for 1 year) to assess whether cumulative effect of smoking affected the association between temperature and lung function. Accounting for smoking pack years and an interaction term between temperature and pack years, temperature remained associated with FEV_1_ (NHANES III *p* = 0.032; NHANES 2007–2012 *p* = 0.007) in survey weighted regressions.

We also assessed whether the association between temperature and lung function was present in subjects with self-reported asthma. Using survey weighted adjusted regressions we found that the magnitude of the effect size was similar to that seen in the general population (NHANES III co-efficient per 10°F = -0.75; NHANES 2007–2012 co-efficient per 10°F = -0.51), but the coefficients were not significant (NHANES III *p* = 0.62; NHANES 2007–2012 *p* = 0.50), which may be a function of being underpowered (NHANES III n = 603; NHANES 2007–2012 n = 1178).

## Conclusions

In separate and pooled analyses of nationally representative samples recruited approximately 20 years apart, there was a statistically significant association between warmer ambient temperatures and reduced lung function. A 30°F difference in mean annual ambient temperature between colder northern regions in the continental United States and the warmer southern regions translates into individuals in warmer regions having lung function that was ~2 percent predicted lower on average than those living in colder regions. To place this in context, the observed effect size is similar to the effect of traffic pollution but more modest compared to that seen with reported tobacco use in both NHANES cohorts (~6 percent predicted lower). Increased traffic exposure near residential addresses has been associated with reduced lung function[25–28] with published estimates of a ~1–3 percent predicted lower measure of FEV_1_.[29,30]

The mechanism(s) leading to this association of lower lung function and warmer ambient temperatures is uncertain. While it is possible that ambient temperature has a direct effect on lung function,[24] the association between mean annual ambient temperatures and lung function did not change when we accounted for the temperature at which spirometry was conducted. One possible indirect mechanism may be the geographic distribution of respiratory pathogens as an increased incidence of respiratory infections in particular regions may lead to lower lung function for the population as whole even if only a limited number of people are affected. Within the general population, limited data on the proportion of MRSA isolates among all *S*. *aureus* isolates suggests that there may be geographical trends in the distribution of the proportion of MRSA isolates from *S*. *aureus* isolates in the United States and Europe. Indeed, certain respiratory pathogens in cystic fibrosis patients (*P*. *aeruginosa*, mycobacteria and MRSA) are more prevalent in warmer climates and partially account for the relationship between reduced lung function and warmer temperatures.[17,15] Alternatively, other indirect mechanisms that may alter lung function include exercise[31] or indoor air pollution,[32,33] which may be subject to regional differences due to outside ambient temperature.[34,35] Additionally, it is possible that a confounding factor could lead to varying lung functions locally. For example, living in an urban area may lead to exposure to a higher mean annual ambient temperature compared to living in the same geographic spot if it were rural, and living in an urban area could also lead to greater exposure to air pollution, which adversely affects lung function.[8,9,36]

There are a number of limitations to this study. First, in a cross-sectional study, there is the possibility of reverse causality, specifically, individuals with health conditions might move to a warm climate because of perceived benefit. However, our exclusion of individuals with asthma or active tobacco use mitigated this possibility. Second, participants may reside in multiple locations over their lives. However, such migration would lead to a conservative bias, i.e. attenuating the extent of a relationship. Another limitation relates to uncertainties in the measurement of temperature. In order to minimize year-to-year variation in temperature, we utilized a 30 year average. The use of an average temperature may underestimate the effect size as average ambient temperature may not reflect what the individual is actually breathing or exposed to. For example, it may be that increased temperatures at certain times of the year (e.g., summer) may have greater effects on health.[37] Other limitations include potential confounding factors that we were unable to assess, such as prior history of tobacco use, physical activity, allergens, and air pollution.[14,38,39] Lastly, while we excluded participants with asthma, some individuals with respiratory conditions were likely included in our analyses, because these respiratory conditions (e.g., COPD and environmental allergies) were not coded. If these individuals were geographically distributed such that certain respiratory conditions were more prevalent in warmer climates, then this could have influenced our results.[40]

Our findings have several clinical and public health implications. Reduced lung function has been shown to be a predictor of mortality in the general population independent of smoking status.[41,42] Published mortality estimates from the Buffalo Health Study incorporating 29 years of follow-up data indicate a 1 to 1.5% decrease in mortality risk associated with a 1 percent predicted increase in FEV_1_,[41] which would potentially correspond to an increase in mortality risk of 2–3% with a reduction of ~2 FEV_1_ percent predicted associated with residing in a warmer region. Of potentially greater medical import is the possibility that individuals with chronic lung diseases may be more susceptible to reduced FEV_1_ as a function of living in warmer regions or with climate change. Several independent samples of individuals with cystic fibrosis in the United States and Australia revealed an association between reduced baseline lung function and warmer mean annual ambient temperatures.[15] Based on these findings, a hypothetical 18 year old white male with cystic fibrosis would have approximately 2.5 percentile point lower lung function associated with every 10°F increase in mean annual ambient temperature. Thus, increased mortality and morbidity with obstructive lung diseases, such as asthma or COPD,[12–14] could arise from the combination of baseline reduced lung function with warmer mean annual ambient temperatures and the additional decrement in lung function with temperature extremes.[43] We would speculate that the reduction in baseline lung function associated with warmer temperatures could be greater in individuals with chronic lung disease than that observed in the general population.

In the context of public health and effects of climate change, the role of temperature assumes additional importance.[11] The mean ambient temperature changes associated with anthropogenic global warming by the end of the 21^st^ century may be 0.3–1.7°C (0.5–3.1°F) under the lowest emission scenario and 2.6–4.8°C (4.7–8.6°F) under the highest.[44] Assuming the lowest emission scenario temperature change (0.3°C) and the lower lung function change with temperature (0.59%/10°F), then the average lung function would be 0.03 percent predicted lower. Conversely, assuming the highest emission scenario temperature change (4.8°C) and the larger lung function change with temperature (0.71%/10°F), then the average lung function would be 0.61 percent predicted lower. Thus, climate change could lead to reduction in lung function ranging from 0.03 to 0.61 percent predicted lower, causing an additional ~340 to 6700 individuals per 1 million to have an abnormal FEV_1_ (<80% predicted).

In conclusion, our findings indicate that warmer ambient temperatures is associated with reduced lung function in the general population. While the observed change of 0.65 percent predicted per 10°F is modest on the population level, it may still lead to an increase in overall respiratory disease burden as it is likely that some individuals may be more susceptible to increases in ambient temperatures, particularly those with chronic respiratory diseases.

## Supporting information

S1 Fig Study exclusions. This flow chart outlines the study populations and relevant exclusions.
(PDF)

S1 Table Included and excluded participants. This table provides demographics for included versus excluded subjects.
(DOCX)
