# Supplementary figures and images for: The relationship of lung function with ambient temperature
Source: PLoS One. 2018 Jan 18;13(1):e0191409. doi: 10.1371/journal.pone.0191409 (PMC5773195; doi:10.1371/journal.pone.0191409)

**Supplemental Figure 1. Study Exclusions**

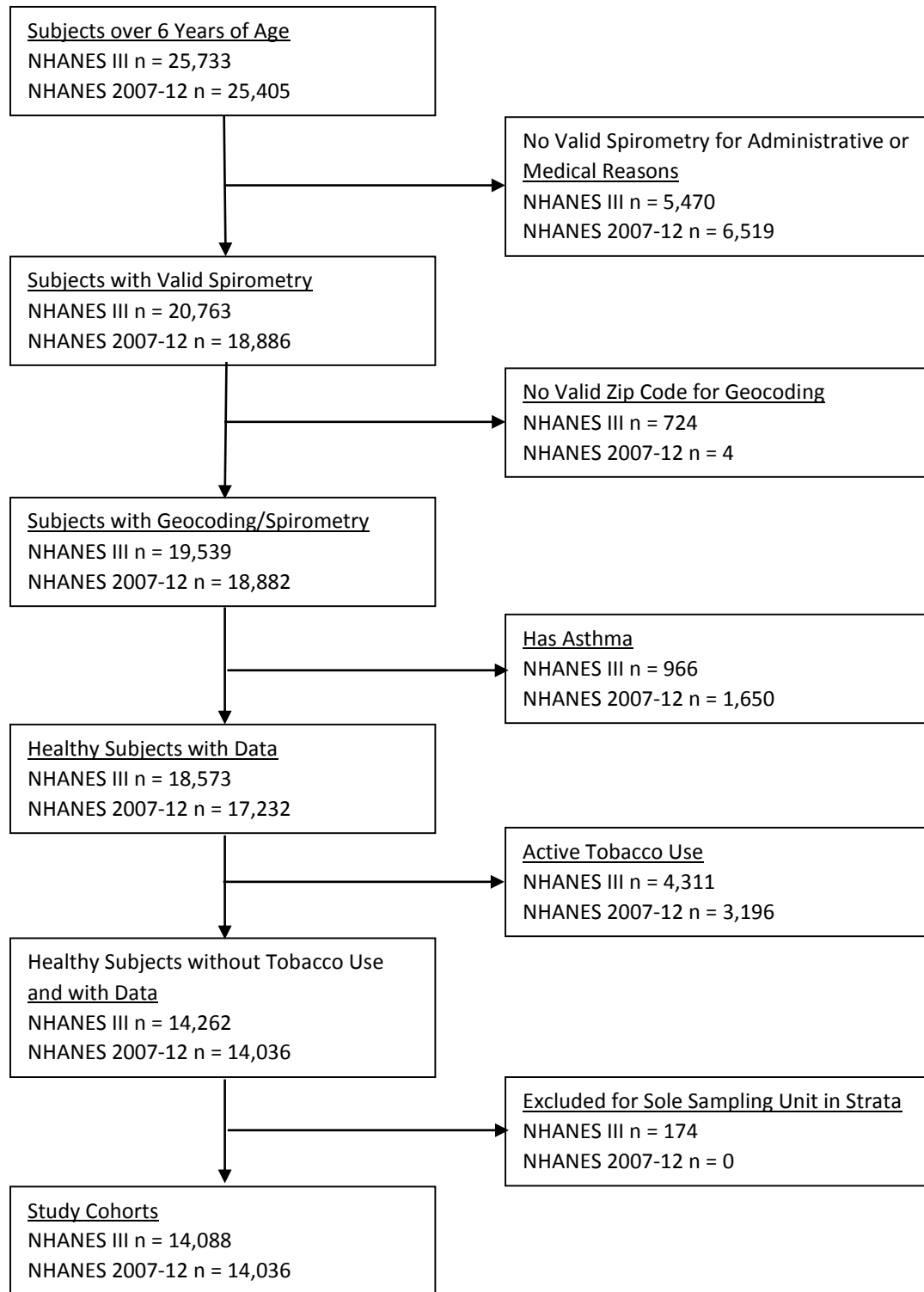

Supplement: S1 Fig — This flow chart outlines the study populations and relevant exclusions. (PDF) [file pone.0191409.s001.pdf]
