# Supplementary material for: The relationship of lung function with ambient temperature
Source: PLoS One. 2018 Jan 18;13(1):e0191409. doi: 10.1371/journal.pone.0191409 (PMC5773195; doi:10.1371/journal.pone.0191409)
Supplement: S1 Table — This table provides demographics for included versus excluded subjects. (DOCX) [file pone.0191409.s002.docx]

**Supplemental Table 1.** Included and Excluded Participants

| Mean ± S.D.  [Range] | **NHANES III Data** | | **NHANES 2007-2012 Data** | |
| --- | --- | --- | --- | --- |
|  | Included Participants  (n = 14,088) | Excluded Participants  (n = 11,645) | Included Participants  (n = 14,036) | Excluded Participants  (n = 11,369) |
| **Sex** (% female) | 55.6 | 49.0 | 52.3 | 47.9 |
| **Race/Ethn.** (%)  Black  Hispanic  White | 27.7  34.1  36.8 | 30.3  26.5  41.4 | 21.7  32.3  35.8 | 24.0  24.8  43.6 |
| Mixed/Other | 1.4 | 1.8 | 10.2 | 7.5 |
| **Age** (years) | 37.4 ± 23.4  [8.0, 90.0] | 41.8 ± 24.4  [6.0, 90.0] | 34.4 ± 21.8  [6.0, 79.0] | 43.1 ± 23.6  [6.0, 80.0] |
| **Income** ($’000s) | 25.5 ± 15.6  [0.01, 50.0]  (n = 12,815) | 23.3 ± 15.4  [0.01, 50.0]  (n = 10,265) | 50.5 ± 32.2  [2.5, 100.0]  (n =13,452) | 40.6 ± 30.2  [2.5, 100.0]  (n =10,750) |
| **Insurance** (%)  None  Private  Public | 17.9  50.8  31.4  (n = 13,497) | 16.6  45.6  37.8  (n = 11,034) | 19.5  53.2  27.3  (n = 14,008) | 22.6  43.4  34.0  (n = 11,352) |
| **Asthma** (% yes) | 0.0 | 11.1 | 0.0 | 20.1 |
| **Tobacco Use** (% yes) | 0.0 | 49.0 | 0.0 | 37.7 |
| **Temperature** (°F) | 60.1 ± 7.8  [41.7, 76.6] | 59.6 ± 7.6  [41.7, 76.6]  (n = 10,710) | 58.4 ± 8.1  [42.1, 76.6] | 57.9 ± 8.1  [42.1. 76.6]  (n = 11,363) |
| **FEV_1_** (% Predicted) | 99.8 ± 15.3  [22.9, 158.7] | 93.3 ± 17.7  [10.6, 149.6]  (n = 6,175) | 99.2 ± 14.5  [23.6, 155.5] | 93.2 ± 16.5  [22.9, 154,5]  (n = 4,850) |
